# Experimental and theoretical study for removal of trimethoprim from wastewater using organically modified silica with pyrazole-3-carbaldehyde bridged to copper ions

**DOI:** 10.1186/s13065-022-00814-0

**Published:** 2022-03-21

**Authors:** Shehdeh Jodeh, Ahlam Jaber, Ghadir Hanbali, Younes Massad, Zaki S. Safi, Smaail Radi, Valbonë Mehmeti, Avni Berisha, Said Tighadouini, Omar Dagdag

**Affiliations:** 1 Department of Chemistry, An-Najah National University, P.O. Box 7, Nablus, Palestine; 2 Chemistry Department, Faculty of Science, Al Azhar University-Gaza, P.O Box 1277, Gaza, Palestine; 3 Laboratory of Applied and Environmental Chemistry (LCAE), Faculté Des Sciences, Université Mohamed Premier, 60 000 Oujda, Morocco; 4 Department of Chemistry, Faculty of Natural and Mathematics Science, University of Prishtina, 10000 Prishtina,, Kosovo; 5 Laboratory of Organic Synthesis, Extraction and Valorization, Faculty of Sciences Ain Chock, Hassan II University, EL Jadida Road, Km 2, BP: 5366, 20100 Casablanca, Morocco; 6 Institute of Nanotechnology and Water Sustainability, College of Science, Engineering and Technology, University of South Africa, 1710 Johannesburg, South Africa

**Keywords:** Global reactivity descriptors, Density functional theory (DFT), Adsorption, Wastewater, Molecular dynamic simulation, Trimethoprim, Pyrazole-3-carbaldehyde

## Abstract

**Background:**

Human and veterinary antibiotics are typically discharged as parent chemicals in urine or feces and are known to be released into the environment via wastewater treatment plants (WWTPs). Several research investigations have recently been conducted on the removal and bioremediation of pharmaceutical and personal care products (PPCPs) disposed of in wastewater.

**Results:**

SiNP-Cu, a chelating matrix, was produced by delaying and slowing 1.5-dimethyl-1H-pyrazole-3-carbaldehyde on silica gel from functionalized with 3-aminopropyltrimethoxysilane. The prepared sorbent material was characterized using several techniques including BET surface area, FT-IR spectroscopy, Scanning electron microscopy (SEM), thermogravimetric analysis (TGA), and nitrogen adsorption–desorption isotherm. The pseudo-second-order model provided the best correlation due to the big match between the experimental and theoretical of different adsorption coefficients. The Langmuir and Freundlich adsorption models were used and the study showed a better match with the Freundlich model with a capacity of removal reached up to 420 mg g^−1^. The removal capacity was dependent on pH and increased by increasing pH. The removal percentage reached 91;5% at pH = 8. The adsorbent demonstrated a high percentage removal of TMP, reaching more than 94% when increased pH. The sample was simply regenerated by soaking it for a few minutes in 1 N HCl and drying it. The sorbent was repeated five times with no discernible decrease in removal capacity. The thermodynamic study also showed endothermic, increasing randomness and not spontaneous. The free energy was 2.71 kJ/mol at 320 K. The findings of the DFT B3LYP/6–31 + g (d, p) local reactivity descriptors revealed that nitrogen atoms and π-electrons of the benzene and pyrimidine rings in the TMP are responsible for the adsorption process with the SiNP surface. The negative values of the adsorption energies obtained by molecular dynamic simulation indicated the spontaneity of the adsorption process.

**Conclusion:**

The global reactivity indices prove that TMP is stable and it can be removed from wastewater using SiNP surface. The results of the local reactivity indices concluded that the active centers for the adsorption process are the nitrogen atoms and the π-electrons of the pyrimidine and benzene rings. Furthermore, the positive value of the maximum charge transfer number (*ΔN*) proves that TMP has a great tendency to donate electrons to SiNP surface during the process of adsorption.

## Introduction

Recently, several research studies for pharmaceutical and maybe personal care products (PPCPs) have been disposed of around the world in wastewater [1]. Human and veterinary antibiotics are typically discharged as parent chemicals in urine or feces and are known to be released into the environment via wastewater treatment plants (WWTPs). Several of the wastewater treatment plants (WWTPs) do not degrade all organic pollutants and this leads to the discharge of the effluent that contains these compounds that causes a major source of environmental pollution. Many organic pollutants can resist treatment in WWTPs in some circumstances, and the range of organic pollutants in WWTP effluents can exceed the mg/L threshold [2]. Also, water pollution from some terminal in thermal power plants during the construction and operation may have some contaminants [3]. Over the last decade, the development of beryllium mining, beneficiation and metallurgy has led to the generation of a large amount of wastewater with high contents Be(II) ions and various Be(NH2)2 complexes; this wastewater has become an urgent environmental issue [4]. In Palestine, when the surface water was sampled, it showed the presence of PPCPs that are environmentally persistent. As we know, trimethoprim (TMP) are used widely for the treatment of both human and veterinary diseases especially in the Middle East and South Africa. The removal efficiencies of secondary treatment for TMP is in the range of 14–87% and it can reach up to 12.5 mg/L in some wastewater treatment plants, especially in the spring season [5].

The presence of those antibiotics in the wastewater and generally in the environment, causes several adverse effects, like bacteria that are resistant to antibiotics [6]. Therefore, several studies on the removal of antibiotics from bodies of water has been conducted [7].

Trimethoprim (TMP) which is critical sulfonamide antibiotic that are frequently discovered in wastewater treatment plants causes more scientists that show interest in how TMP antibiotics is biodegraded [8]. One study used prepared composite soil to allow biodegradation to successfully remove adsorbed TMP from the surface of clay ceramists.

Several methods are known for removal of antibiotics from wastewater like ion exchange, co-precipitation, and liquid–liquid extraction [9]*.* These methods often demand a large amount of high purity organic solvents, which can be dangerous to health and cause environmental problems.

For sample treatment, some procedures are used, such as solid phase extraction (SPE), which was utilized for the pre-concentration/separation of a wide range of inorganic and organic substances [10].

To remove trace metal ions, and antibiotics from wastewater, various ligands or functional groups are occasionally attached to a solid that serves as a solid phase extraction matrix. Several researchers use silica [11–13]. The use of silica is becoming increasingly important in today's world. Silica is a major component of the modern industrial base, and it is used in a variety of industries ranging from glass manufacturing to oil extraction. Silica has several benefits, including a large surface area and good mechanical and thermal stability. Also, by interacting with organofunctionalized silanes groups, it is simple to modify and functionalize the structure [14, 15]. These covalently bonded chemical groups serve as a highly stable long arm spacer, allowing the receptor to make contact with the copper ion and then TMP. The chelating material was described and its adsorption capacities for removing TMP from aqueous media were investigated [16]. The advantages of them include the ability to operate without losing expensive organic molecules, which is due to the nature of grafted ligands, which can attach molecules with chelating ability due to their characteristics of donor atoms, such as nitrogen, oxygen and sulfur [17]. This paper described the previously synthesized and characterized some silica that has been functionalized with some compounds such as N,N′-bidentate, resulting in the formation of five-membered chelating rings [18]. The copper-ion-attached compound was employed to remove TMP from the produced aqueous solution. Even several research studies for pharmaceutical and personal care products (PPCPs) have been occurring around the world, the use of 1,5-Dimethyl-1H-pyrazole-3-carbaldehyde, which was fixed on the silica surface after many treatments and modifications, including 3-aminopyltrimethoxysilane, and then refluxed with copper nitrate to produce SiNP-Cu made it very novel for this application with very high efficiency of removal and regeneration. In addition to that, theoretical studies were very supportive of our study.

## Experimental

### Chemicals and instrumentations

Trimethoprim (TMP), all chemicals and solvents that have been used in this study including silica gel and the silylating agent were of high purity > > 99.5%, Where bought from Sigma-Aldrich, Saint-Louis, MO, USA. Before using silica, it was always activated by heating for roughly 24 h at 160 °C.

**Fig. 1:**
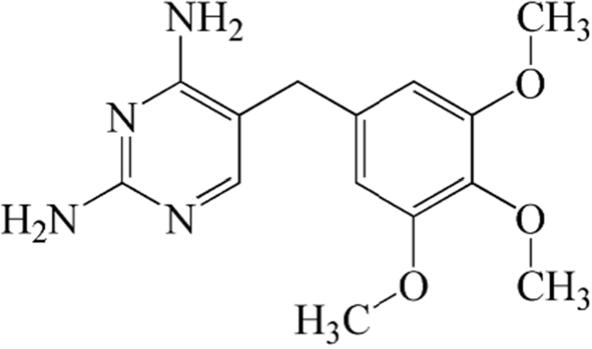
Molecular structure for trimethoprim (TMP)

The formula of TMP was shown in Fig. 1. To increase the solubility of TMP certain proportion of ethanol and distilled water were used.

Several instruments were used for this research including shaking water bath (Daihan Labtech, Korea), with variable speed of 20 to 500 rpm to enhance mixing of both adsorbate and adsorbent, thermometers, pH meter (model: 3510, JENEWAY, USA) to be used for the effect of pH study, Infrared spectroscopy (FTIR-SHIMADZU, Japan, Model: FTIR-8700). FT-IR Spectra of SiG, SiNH_2_, and SiNP spectral were obtained with a resolution of 2 cm^−1^ over a range of 1 cm^−1^ to 4000 cm^−1^. Scanning Electron Microscopy (SU8000 Hitachi, Japan). The specific area was determined by using the BET equation. Brunauer–Emmett–Teller (Micromeritics, Norcross, GA, USA), The TGA Instrument was used to determine the mass loss (TGA Instruments, New Castle, DE, USA) from 20 to 900 °C at a rate of 10 °C min^−1^. All experimental details are explained well in our previous study.

### Synthesis of 3-aminopropylsilica (SiNH2)

**Scheme 1:**
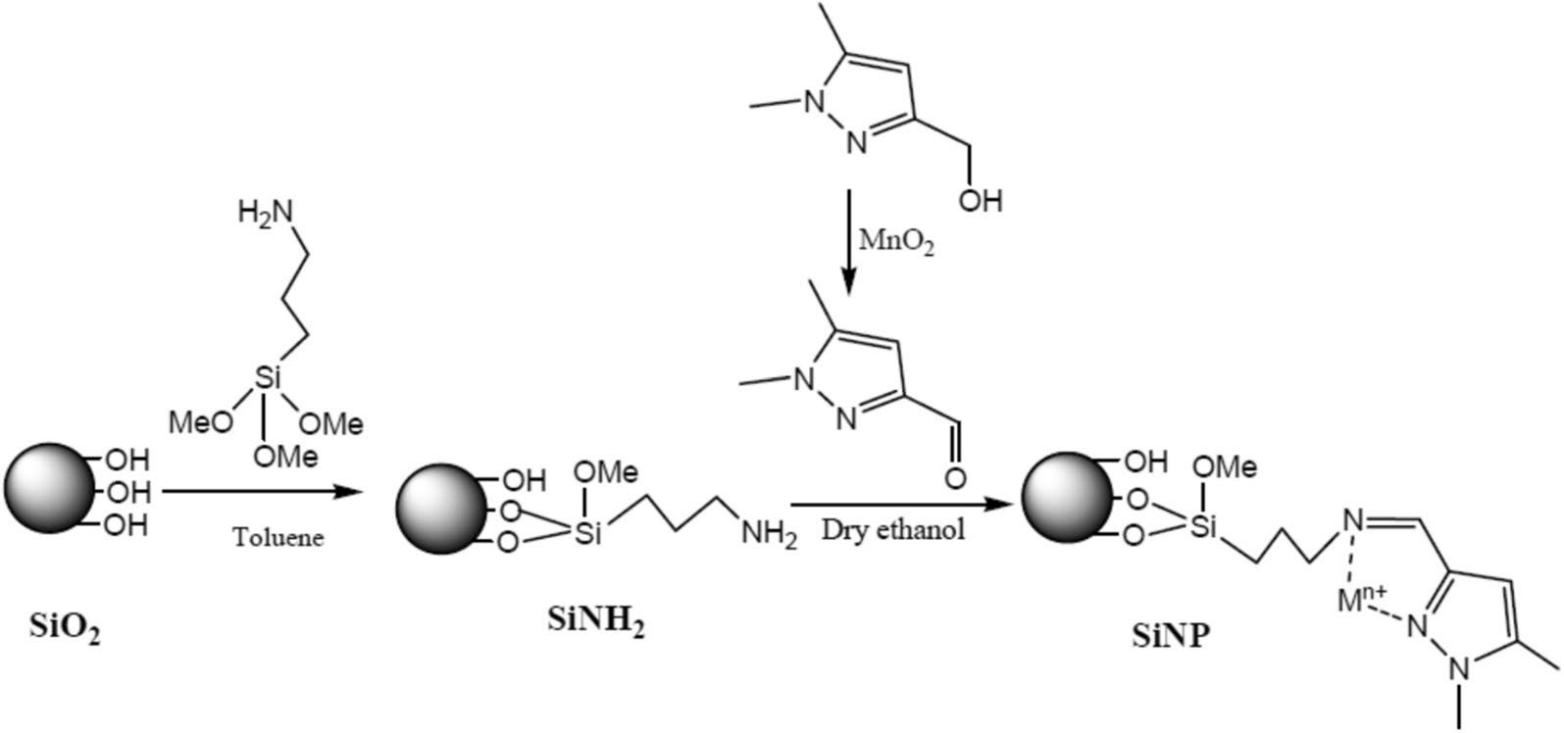
Modified chelating material synthesis scheme

As mentioned before this material has been synthesized before and used for removal of metal ions [19] with a small modification of attaching copper to SiNP and to be referred as SiNP-Cu. In summary, the first step was to carry out a reaction on the silica surface between the silylating agent and silanol groups. 25 g of activated silica gel (SiO_2_) was added to 150 mL of toluene and refluxed for 2 h under a nitrogen environment with constant stirring. The preceding solution was then treated with 10 mL of aminopropyltrimethoxysilane. Following that, filtering was performed and washed with toluene and ethanol. To remove the silylating residue, a Soxhlet extraction with a mixture of 1:1 ethanol and dichloromethane was done for 12 h. The name of the final product is called SiNH_2._ (Scheme 1).

### Synthesis of 1.5-dimethyl-1H-pyrazole-3-carbaldehyde

A 1.2 g of (1.5-Dimethyl-1H-pyrazol-3-yl) methanol was dissolved in 100 mL of dioxane. After that, an amount of manganese oxide was added and refluxed for about 5 h [20]. The solution was filtered and MnO_2_(s) was removed.

### Synthesis of ((1,5-Dimethyl-1H-pyrazol-3-yl) methylene) imine-substituted silica (SiNP)

To prepare the above mixture, a 5 g of the solution prepared in step 2.2 (SiNH_2_) and 1.5 g of the solution prepared in step 2.3 were mixed in dry ethanol. The solution was filtered and soxhlet extracted with methanol and acetonitrile. This product is referred to as (SiNP).

### Synthesis of SiNP-Cu

A 1 g of the product of step 2.4 (SiNP) was mixed with 1.5 g of copper nitrate in 100 mL dry ethanol and then Soxhlet extraction with acetonitrile for 2 h. The mixture was washed several times with ethanol and dried. The final product was checked with the Thermo Scientific™ iCE™ 3300 AAS flame atomic absorption to check for copper.

### Batch experiments

Using a 0.1 g of (SiNP-Cu) and 25 mL of TMP solution at various concentrations, the effects of contact time (0–150 min), pH (2–12), dose (0.01–0.3 g), and temperature were investigated.

To achieve the appropriate pH, a solution containing both NaOH and HCl was used. To aid interaction between adsorbent and adsorbate, all flasks were capped and shaken using a water bath at a specified temperature and 150 rpm. A portion of the supernatant was obtained after 40 min and centrifuged mechanically at 4000 rpm for 10 min before being tested for TMP using the Shimadzu UV–Visible 1601 model at a wavelength of 272 nm. A stock solution of TMP was produced using 1L deionized water at room temperature (25 °C) for data analysis. The adsorbent is washed with 0.1 N HCl solution and then with distilled water after each adsorption process. Following that, each regenerated adsorbent is allowed to dry for 24 h before being used again to demonstrate that the prepared adsorbent can be used several times with little effect on the percent removal of TMP. To study other parameters like kinetics, isotherm, and thermodynamics, the same procedures were used but a range of different temperatures (310, 315, and 320 K) were used. In our study, each parameter's data was analyzed twice, and only the mean data was published and plotted.

### Computational details

#### DFT part

The Gaussian 09 package was used to perform all density functional theory (DFT) computations [21]. TMP’s geometry was optimized without constraints utilizing the B3LYP exchange- correlation functional level [22, 23]. For this project, the 6–31 + g (d, p) basis set was used [24]. The polarizable continuum model (PCM) solvation approach was used to calculate the solvation effect using the self-consistent reaction field (SCRF) method [25–27]. At the same level, frequency calculations were used to represent the stationary points and confirm that the ground states do not have an imaginary frequency. Some of the energy gaps can be closed by using the optimized structure's highest occupied molecular orbital energies (EHOMO) and lowest unoccupied molecular orbital energies (ELUMO) ($\Delta E= {E}_{LUMO}- {E}_{HOMO}$), electronic chemical potential ($\mu =\frac{1}{2}\left({E}_{LUMO}+ {E}_{HOMO}\right)),$ global hardness (η = $\frac{1}{2}\left({E}_{LUMO}-{E}_{HOMO}\right))$, hyper-hardness (γ = ${E}_{LUMO}-2{E}_{HOMO}+ {E}_{HOMO-1}$), softness (S = $1/\eta$),, electrophilicity (*ω* = ${\mu }^{2}/2\eta$) and maximum charge transfer (ΔN = $-\mu /\eta$) are calculated for TMP molecule [28, 29].

The Fukui functions (FF) were used to compute the local reactivity descriptors (LRD) of TMP for nucleophilic attacks ($${f}_{k}^{+}={q}_{k}\left(N+1\right)-{q}_{k}\left(N\right))$$ and for electrophilic attacks ($${f}_{k}^{-}={q}_{k}\left(N\right)-{q}_{k}\left(N-1\right)$$) [30, 31]. The charges values of atom (k) for neutral, cation, and anion species are denoted by$${q}_{k}(N)$$, $${q}_{k}(N+1)$$ and $${q}_{k}(N-1)$$ respectively. These indices numerically signify the most reactive centers that are responsible for the interactions with SiNP surface. Additionally, the local softness ($${\sigma }_{k}^{\alpha }=s{f}_{k}^{\alpha })$$ and local electrophilicity ($${\omega }_{k}^{\alpha }=\omega {f}_{k}^{\alpha }$$) were also calculated [32]. The letter *α* = (+) and/or (−) describes the nucleophilic and electrophilic attacks, respectively. More precisely and to get a clearer identification for the electrophilic and the nucleophilic attack at specific atomic sites [33, 34], the dual descriptors (DDs) (Fukui descriptor or the second order Fukui functions ($${f}_{k}^{2}= {f}_{k}^{+}-{f}_{k}^{-})$$, dual softness ($$\Delta {\sigma }_{k}={\sigma }_{k}^{+}-{\sigma }_{k}^{-})$$ and the dual philicity $$\Delta {\omega }_{k}={\omega }_{k}^{+}-{\omega }_{k}^{-}$$) were also calculated. These DDs simplify chemical reactivity in a local sense and allow us to obtain the prefered locations for nucleophilic attacks and the preferred sites for electrophilic attacks ($${f}_{k}^{2} ,\Delta {\sigma }_{k} and \Delta {\omega }_{k}$$< 0) [35].

## Results and discussion

### Characterization

#### FT-IR characterization

**Fig. 2:**
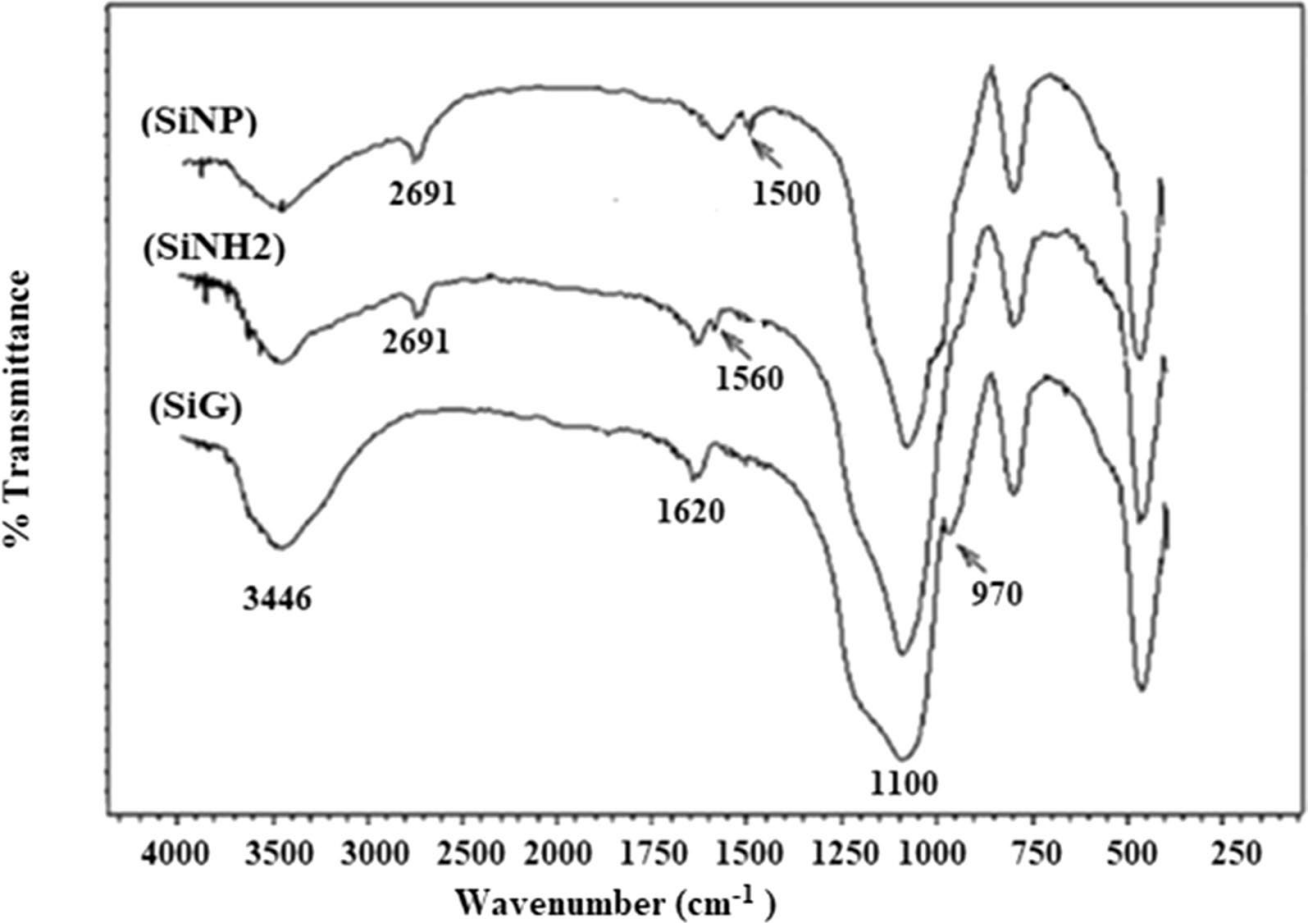
FT-IR Spectra of SiG, SiNH_2_ and SiNP

A Perkin–Elmer FT-IR spectrophotometer (Spectrum BX-II) was used to record FT-IR spectra on KBr disks with a range of 4000–400 cm^−1^ as shown in Fig. 2.

To study the synthesis of new materials that have been synthesized for TMP removal (SiNP-Cu) it is necessary to study the functional groups.

The two peaks observed at 1100 and 3446 cm^−1^ were attributed to Si–O–Si stretching vibrations, and the presence of –OH group. Moreover, the peak observed at 1500 cm^–1^ was related to the C=N vibrations and N–H bending vibration. This verifies the functionalization onto the silica surface [36].

#### Scanning electron micrographs

Scanning electron micrographs (SEM) of SiNP and SiNP-Cu were obtained using a scanning electron microscope (Jeol JSM 60) and an accelerating voltage of 20 kV.

**Fig. 3:**
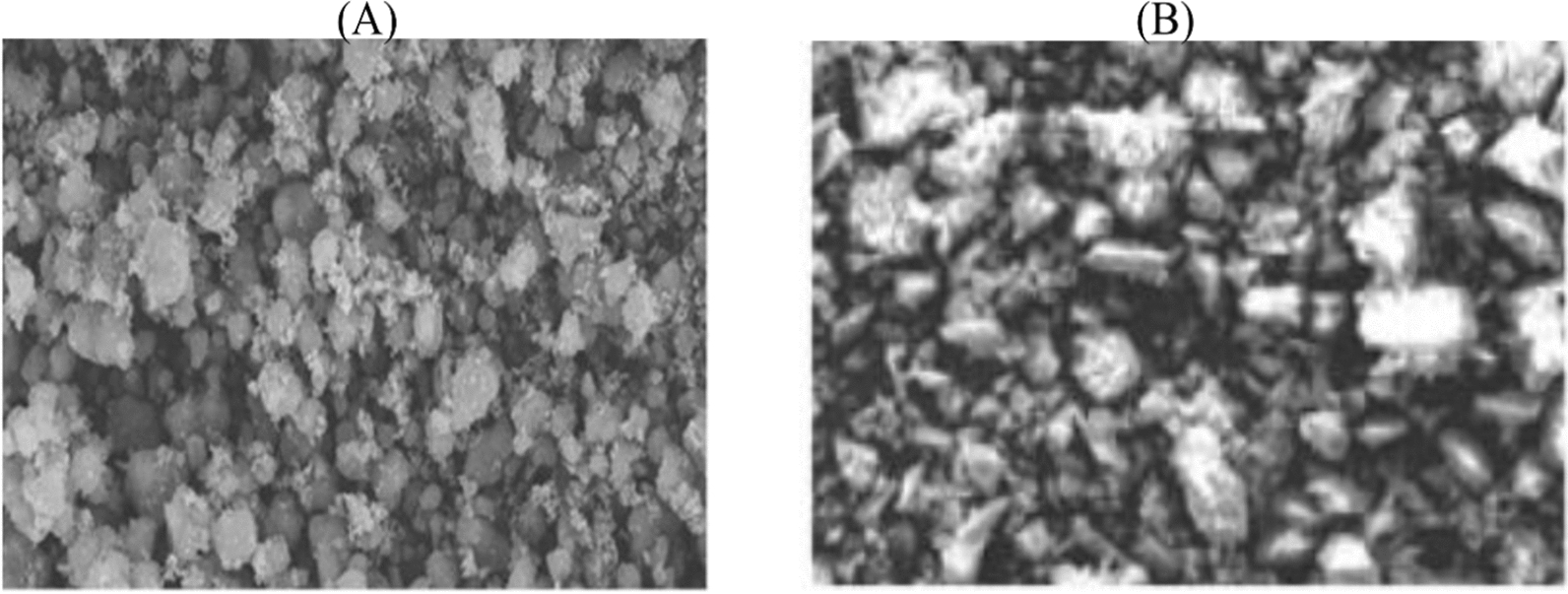
SEM images of SiNP (**A**), SiNP-Cu (**B**) with ×200 magnification

The SEM is shown in Fig. 3 and was obtained at 200× magnification. It is obvious that before SiNP linked with Cu ions the adsorbents exhibit heterogeneous surfaces of high roughness. Also, considering the SiNP-Cu after linkage, many of the pores on the adsorbent surface are well-covered by the Cu ions [37].

#### TGA analysis and thermal stability

**Fig. 4:**
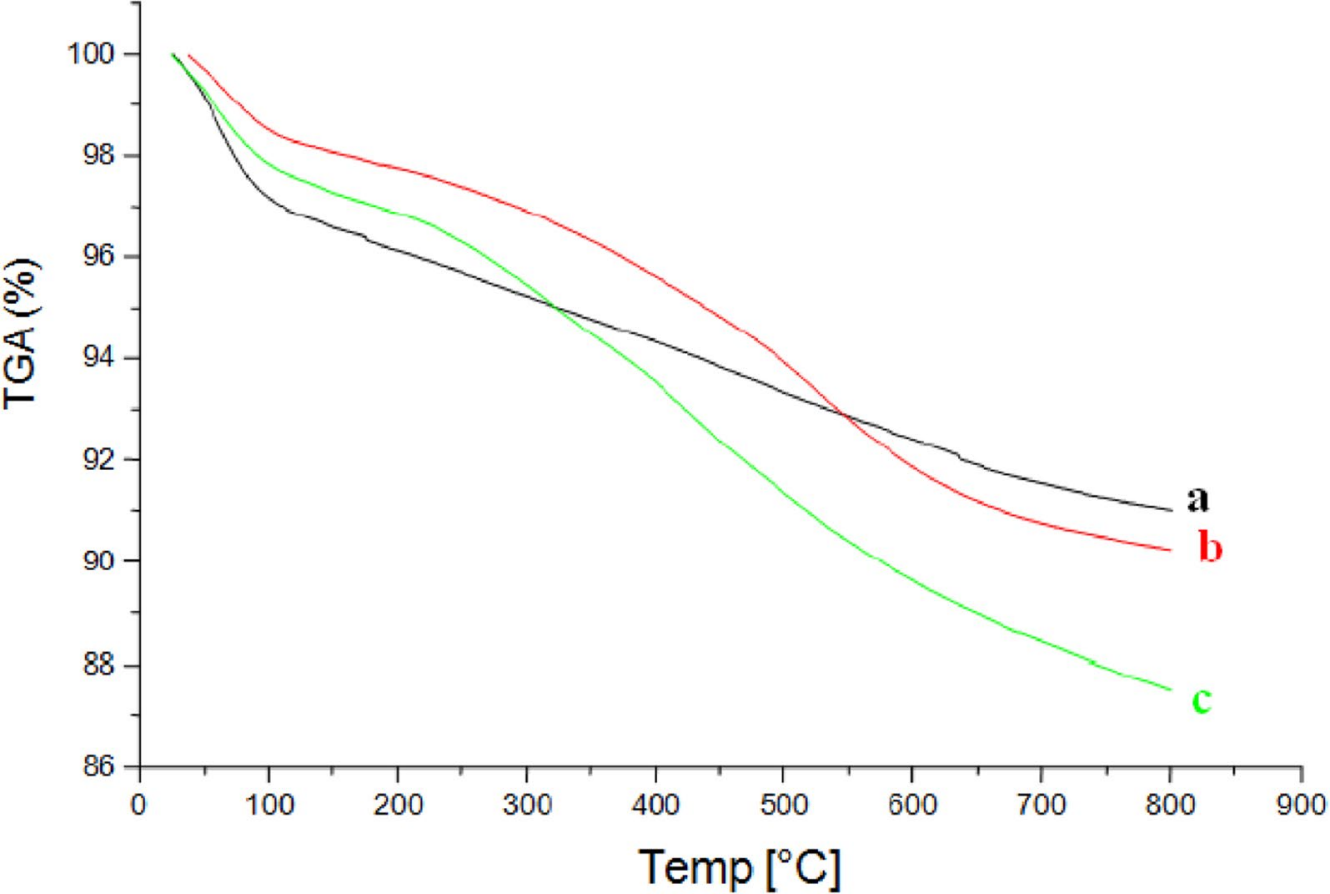
TGA analysis of free silica (**a**), of SiNP (**b**), SiNP-Cu (**c**)

For mass loss and stability studies, a dry sample was heated in nitrogen gas at a rate of 10 °C/min (flow rate: 50 mL/min) as shown in Fig. 4.

This study will help us to have an idea about the surface stability and at the same time to be sure there is mobility on the surface and how much. According to the profiles in a and c, the degradation process occurred between 120 and 800 °C, demonstrating that both SiNP and SiNP-Cu produced materials had high thermal stability.

In the temperature range of ambient temperature to 105 °C, both samples revealed a first mass loss stage of 3.20 percent, which was attributed to the water loss that the samples had absorbed. The second loss was 5.95 percent between 105 and 800 °C which represents the condensation of free silanol groups (Si–O–Si) [38]. The significant rise in mass loss demonstrates the presence of a large number of anchoring organic groups. Again two distinct mass loss steps were detected for the SiNH2 sample. The first one, a small mass loss of 1.56% in the room temperature to 100 °C range is attributed to the remaining silanol hydration water, as a consequence of the use of these groups in the immobilization process. On the other hand, a pronounced mass loss increase of 9.77% was observed for the second step, between 208 and 800 °C, which corresponds to the organic matter added onto the surface during immobilization. The final SiNP material presented two distinct mass loss stages.

#### Surface properties

The nitrogen adsorption isotherm was used to characterize the produced compounds' surface area, pore sizes, and volume. The pore diameter was calculated using the Barrett-Joyner-Halenda (BJH) method [39]. The pore volume was 0.77 cm^3^/g and the surface area was 310 m^2^/g.

#### Batch method

To study the effects of pH, contact time, the effect of dosage and temperatures, A 10 mg of the synthesized material (SiNP-Cu) was added to mL of the TMP solution and shaking for a period of time. The contact time was studied up to 150 min. The range of pH was from 2 to 12, the dosage was from 0.01 to 0.3 g. Three different temperatures were used during this study (310, 315 and 320 K). The concentration of TMP was determined by spectrophotometry measurements. The amount of TMP removed by the prepared material SiNP-Cu from the aqueous medium was calculated using the following equations [40]:

1 $$Q_{M} = \frac{{\left( {C_{0} - C_{e} } \right)}}{W}$$

2 $$Q_{W} = Q_{M} \times M$$

Q_M_ denotes the quantity of TMP on the adsorbent (mmol/g), Q_W_ denotes the amount of TMP on the adsorbent (mg/g), V denotes the volume of the aqueous solution (L), W denotes the weight of the adsorbent (g), and C_0_ denotes the initial concentration of TMP (mmol/L), The equilibrium concentration of TMP in the prepared solution (mmol/L) is represented by C_e_, while the atomic weight of TMP (g/mol) is represented by M. Analyses were carried out in duplicate for each sample, and the mean data is what is provided.

### Sorption experiments

#### Effect of adsorbent dose

**Fig. 5:**
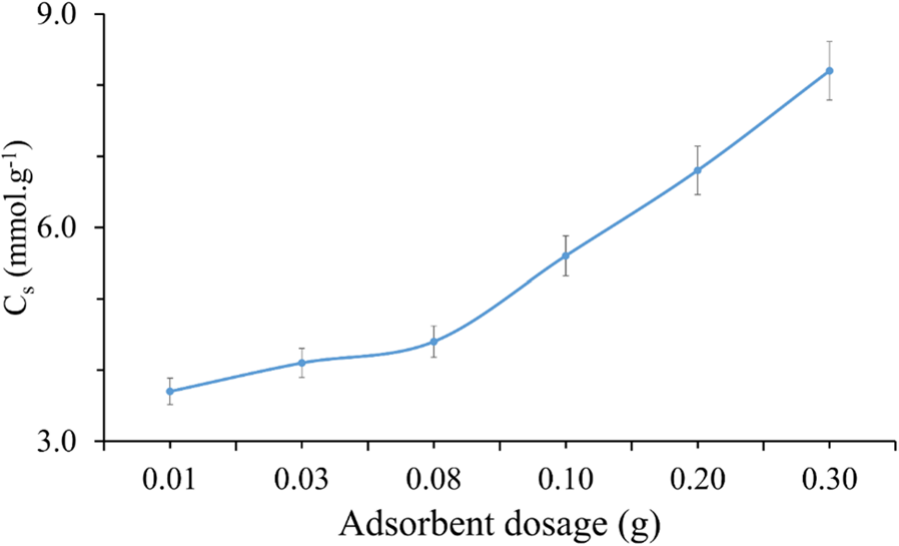
Effect of adsorbent dosage on removal TMP by SiNP-Cu using 50 mL of 50 mg/L of TMP at pH = 8 and room temperature

Several parameters were studied to determine the capacity of an adsorbent. One of them is the effect of adsorbent dose on adsorption of TMP by SiNP-Cu which showed a higher removal capacity of TMP during the increasing of the dose amount as shown in Fig. 5. This rise is due to an increase in the number of available reaction sites for the adsorbent. With 300 mg of SiNP-Cu, about % of TMP was removed from a 50 mL solution.

#### Effect of pH

Previous research has shown that the adsorption of medicines to functionalized compounds in solution or placed on solid supports is usually influenced by numerous parameters such as pharmaceutical compound size, charge, shape of the donor atom [41], as well as their binding properties and buffering conditions [42]. These characteristics were examined utilizing solution chemistry and solid-phase extraction of various materials based on the coordination of the immobilized on the surface of solid supports, such as silica gel, nanomaterials, and polymeric compounds. To investigate the suitability of synthesized SiNP-Cu for TMP removal, the effect of pH was investigated as one of the critical parameters.

**Fig. 6:**
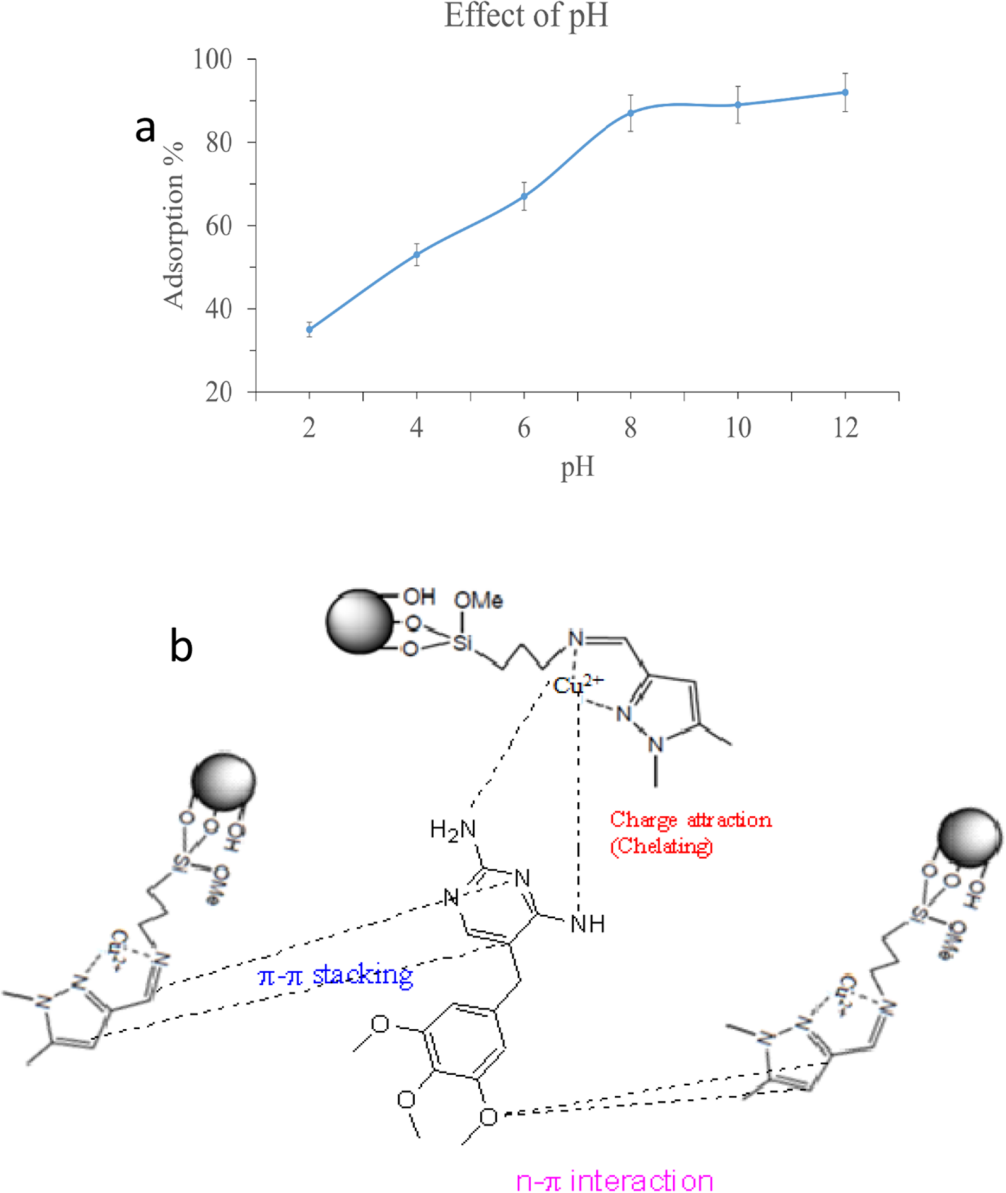
Effect of pH on TMP removal using 100 mg of SiNP-Cu and 50 mL of 50 mg/L solutions at room temperature (**a**), TMP Adsorption mechanism on SiNP-Cu (**b**)

The adsorption properties of SiNP-Cu were investigated between 2.5 and 11.0 is represented in Fig. 6.

As seen in Fig. 6, the TMP uptake of the adsorbent changes as the pH changes was studied using 50 mL of 50 mg/L solution of TMP at room temperature and 100 mg of SiNP-Cu. The retention of TMP by the functionalized silica SiNP-Cu is not excessive at low pH levels, which is due to the ligand, which must be in its protonated form. When the pH rises, protonation weakens, which improves the chelation and adsorption of pollutants such as TMP.

Figure 6 clearly shows that an increase in pH had an effect on the adsorbent surface and TMP. At pH = 12, the elimination percentage nearly reached 90%. The explanation for this is that TMP is a weak base with a pKa of 7.3. As a result, all TMP take the form of $TH^{ + } \leftrightarrow T: + H^{ + }$.

Another fact is that the protonated form of TMP does not favor the adsorbent surface of the produced SiNP-Cu, which has a positive charge. At acidic pH values, both the TMP and the adsorbent surface are positively charged, which may explain why TMP adsorption on SiNP-Cu surfaces is low. On the other hand, as pH rises, the proportion of adsorption rises as well. In general, the removal percentage reached about 91.5% at pH = 8.

#### Effect of contact time

**Fig. 7:**
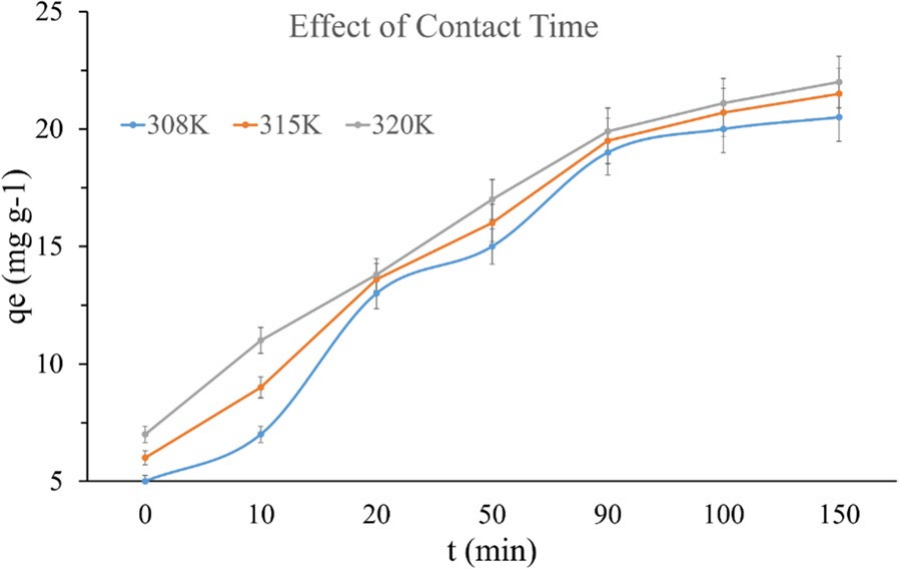
Effect of contact time of TMP adsorption onto SiNP-Cu

At different temperatures, (308, 315, and 320 K), the influence of contact time on TMP adsorption by SiNP-Cu was investigated as seen in Fig. 7. As seen it took 90 min to start reaching the adsorption equilibrium. This time was used for the rest of the batch studies including the effect of dosage and temperatures. The equilibrium times were found to be the same for all temperatures, and they increased as the temperature increased, as seen in the graph.

#### Adsorption kinetics

To study the kinetics for the adsorption and to understand the change in adsorption with time, three models have been applied [43]:

Pseudo-first-order, pseudo-second-order, and intraparticle diffusion models are among them.

Lagergren's pseudo-first-order kinetic model has the following equation:

3 $$\frac{1}{{q_{t} }} = \left( {\frac{{k_{1} }}{{q_{1} }}} \right)\left( \frac{1}{t} \right) + \frac{1}{{q_{1} }}$$

The parameters in the equation are: q_t_ represents the quantity of TMP adsorbed (mg/g) on SiNP-Cu at different times t; q_1_ represents the maximal adsorption capacity (mg/g); and k_1_ represents the pseudo-first-order adsorption rate constant (min^−1^). The parameters (q_1_ and k_1_) were calculated using the intercept and slope of a simulated first-order straight line. Table 1 summarizes these parameters and correlation coefficients (R^2^).

**Table 1:** TMP adsorption onto SiNP-Cu kinetic parameters at various temperatures

Kinetic Model			
Temperature (K)	308	315	320
Pseudo-1st Order			
R_1_^2^	0.75	0.81	0.91
K_1_ (min^−1^)	3.91	6.35	7.32
q_1_ (mg g^−1^)	19.38	19.82	20.3
q_e_ (Calculated)	9.32	11.23	13.62
Qe(exp)	41.21	23.17	48.23
Pseudo-2nd Order			
R_2_^2^	0.992	0.981	0.999
K_2_ (g mg^−1^ min^−1^)	0.003	0.004	0.005
q_2_ (mg g^−1^)	39.32	20.38	40.46
q_e_ (Calculated)	42.86	22.23	47.85
Intra-particle diffusion			
R_p_^2^	0.999	0.964	0.999
K_i_ (mg s^−1\2^ g^−1^)	0.18	0.26	0.19
C	12.32	14.8	15.7

From Table 1, the values for (R_1_^2^) were between 0.75 and 0.91 for the TMP at 308, 315 and 320 K, respectively.

The equation below was used to calculate the parameters for the pseudo-second-order adsorption kinetic rate [44]:

4 $$\frac{{dq_{t} }}{dt} = k_{2} \left( {q_{2} - q_{t} } \right)^{2}$$

For the pseudo-second-order adsorption kinetic model, K_2_ is the rate constant (g mg^−1^ min^−1^) and q_2_ is the maximal adsorption capacity (mg g^−1^). The equation below was generated by integrating the equation and using a boundary condition (q_t_ = 0 at t = 0 and q_t_ = q_t_ at t = t), the equation below was obtained:

5 $$\frac{1}{{q_{t} }} = \frac{1}{{k_{2} d_{2}^{2} }}{ + }\frac{t}{{q_{2} }}$$

A plot of (t/q_t_) versus t was obtained to calculate the parameters that are listed in Table 1. We found that the correlation coefficients (R^2^) of the first-order and pseudo-second-order kinetic models are substantially lower in the first order model than in the second order model by comparing the correlation coefficients (R^2^) of the two models. In addition, using different plots for both kinetic models to compare both calculated and experimental qe, we found that the results for the pseudo second order model correspond better, as shown in Table 1. The second-order kinetic model can better characterize the sorption process for TMP on SiNP-Cu based on the data and results in the Table. Another finding showed that when the temperature rose, the maximum adsorption capabilities of TMP adsorption onto SiNP-Cu increased. This leads to the conclusion that TMP promotes adsorption onto SiNP-Cu. Another finding is that the adsorption process can be regarded as chemical, and TMP adherence occurs from the bulk phase to the solid phase (SiNP-Cu) as the temperature of the solution rises. This finding was also discovered by investigating the isotherm adsorption section.

The study of the intraparticle diffusion model of Weber and Morris can be presented by the mathematical equations [45, 46]:

6 $$q_{t} = k_{t} {\text{t}}^{1/2} {\text{ + C}}$$

**Fig. 8:**
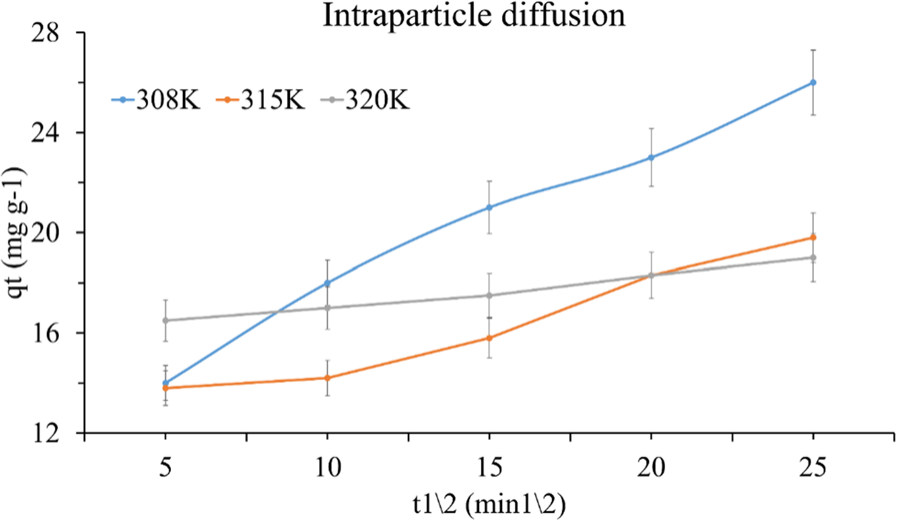
The intraparticle diffusion plot for adsorption of TMP onto SiNP-Cu at different temperatures

In this equation, qt is the quantity of TMP adsorbed (mol/g) at time t, and C is the intercept, which is used to define the thickness of the boundary layer; the larger the intercept, the stronger the boundary layer effect. The intraparticle diffusion rate constant is denoted by k_i_ (mg s^−1^ g^−1^). The slope of qt versus t^1/2^ was used to calculate k_i_. The plot may show multi linearity, indicating that a few steps occur. For the two temperatures (308 and 315), the first portion (14 to 20.5) describes the diffusion of adsorbate from the solution to the adsorbent's external surface or, in some cases, displays the boundary layer diffusion of dissolved molecules. The second portion (20.5 to 23) typically reflects the progressive rise of the layer adsorption stage where intraparticle diffusion is the rate limiting phase. The third portion (22 to 26) is attributed to the ultimate equilibrium stage, as seen in Fig. 8 and Table 1.

Figure 8 shows that the figure for qt vs t0.5 at 320 K is almost straight line, which can demonstrate and establish intraparticle diffusion effects.

As previously stated, the intercept (C) values often describe the thickness of the boundary layer. The bigger the intercept, the stronger the boundary layer effect. In our experiment, the values for C increased with increasing temperature. This demonstrated that the boundary layer effect played no significant influence in the adsorption of TMP onto SiNP-Cu.

Furthermore, the linear sections of the intraparticle diffusion curves in the figure did not pass through the origin, implying that additional effects and processes control the entire adsorption process.

Removal of contaminants in aqueous systems through reverse osmosis, ion exchange, and electrolysis is more expensive compared to when removal proceeds via the adsorption process. Adsorption is an effective and low-cost technique for the elimination of antibiotics in aqueous systems [47].

Application of commercial activated carbons (ACs) for the treatment of wastewaters contaminated with antibiotics has been reported in various studies [48–50]. ACs are universal and more effective to eliminate various pollutants from solution. However, these ACs do not find global acceptance due to their high costs [51].

The physicochemical properties, adsorption capacities, isotherms, and kinetics models of the adsorbents used for the removal of trimethoprim are presented in Table 2. A most recent study done used modified silica to uptake TMP [52]. This adsorbent has been applied as a new promising adsorbent for the uptake of trimethoprim, as the graphene oxide and its derived nanomaterials are known to have outstanding adsorption performance toward antibiotics [52].

**Table 2:** Physicochemical characteristics, adsorption capacities, isotherms and kinetics models of the adsorbents used for removal of trimethoprim

Adsorbent	Surface area (m^2^ g^−1^)	pH	Maximum adsorption capacity (mg g^−1^)	Isotherm model	Kinetic model	References
Micro-AC	1534	6.22	543	Langmuir	Pseudo-second-order model	[48]
Biochar	8.89	9.1	2.08 × 10^3^	Langmuir	Pseudo-second-order model	[49]
Graphene oxide	–	–	204.08	Freundlich	Pseudo-second-order model	[50]
Bentonite	23	8.1	106.27	Langmuir	Pseudo-second-order model	[51]
SiNP-Cu	–	8	420	Freundlich	Pseudo-second-order model	

#### Adsorption isotherms

**Fig. 9:**
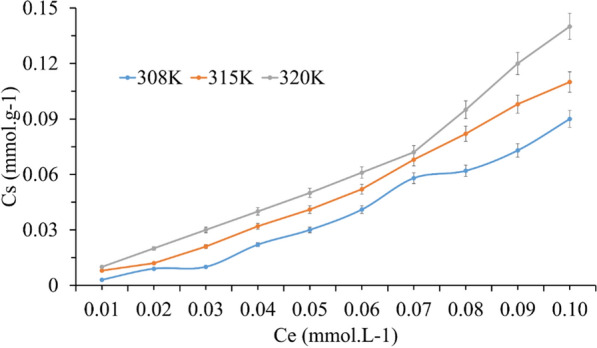
Adsorption isotherms for the TMP onto SiNP-Cu at 308, 315 and 320 K

The results of the change in the adsorbed amount of TMP with equilibrium concentrations on the surface of SiNP-Cu was given in Fig. 9. The symbol C_s_ is representing the solid phase concentration (mmol g^−1^) and the C_e_ symbol is representing the final concentration (mmol L^−1^) in the supernatant during equilibrium for every single initial concentration.

Plotting C_e_/C_s_ vs C_e_, the slope and the shape of the initial portion of these isotherm curves for the three temperatures plot is very close to S-type and C-type as in Giles classification, respectively (Giles, MacEwan et al. 1960). This type of isotherms is relatively rare and indicative of weak adsorbent–adsorbate interactions [53].

Several isotherm equations have been developed and used to study the equilibrium nature of adsorption processes. In our study, we used two models to describe the isotherm equilibrium: The Langmuir and the Freundlich sorption isotherm.

According to Langmuir isotherm principle, it assumes the presence of monolayer coverage of adsorbate over a homogenous adsorbent surface like TMP on SiNP-Cu in our case the adsorption data were represented in Table 3 and were obtained using the linear form of Langmuir adsorption model (Eq. (7)).

7 $$\frac{{C_{e} }}{{C_{s} }}{ = }\frac{1}{{C_{m} a_{L} }}{ + }\frac{{C_{e} }}{{C_{m} }}$$

**Table 3:** Isotherm coefficients for TMP adsorption onto SiNP-Cu at various temperatures

Isotherm Model			
Temperature (K)	308	315	320
Langmuir model			
R^2^	0.71	0.85	0.73
C_m_ (mmol g^−1^)	− 0.02	− 0.03	− 0.18
L (g L^−1^)	− 6.32	− 4.67	− 4.13
R_L_	4.28	2.16	2.32
Freundlich model			
R^2^	0.95	0.98	0.95
K_f_ (mg^(1–1/n)^ g^−1^ L^1/n^)	15.82	8.75	4.75
n_f_	2.11	2.32	1.97

The variables C_s_ and C_e_ were mentioned above while C_m_ is the amount of drug that is required for making monolayer (adsorption capacity). The variable a_L_ is a Langmuir constant and representing the intensity of the adsorption and adsorption energy.

The dimensionless constant separation factor R_L_ was used to study the essential characteristics of the Langmuir isotherm:

8 $$R_{L} { = }\frac{1}{{\left( {1 + a_{L} .C_{0} } \right)}}$$

C_0_ is the initial TMP concentration (mmol L^−1^) and a_L_ is Langmuir constant. All the variables values are summarized in Table 3. Usually, the R_L_ values between 0 and 1 indicate favorable adsorption of TMP onto SiNP-Cu at the concentration studied.

From Table 3 all the R_L_ values at different temperatures were higher than 1. Which indicates that unfavorable adsorption was occurred and also all the adsorption capacity (C_m_) values were negative for the SiNP-Cu. These results agree with a previous study by Molu and Yurdakoc, 2010 [54].

From Table 3, the correlation coefficients (R^2^) obtained for Langmuir equation were smaller than the one obtained by Freundlich equation (around 0.75) which suggested that the boundary layer thickness that has been studied by intraparticle diffusion was also increased. The mathematical Freundlich equation (Eq. (9)) is an exponential equation that usually used for multilayer adsorption with a heterogeneous energy distribution of active sites. The equation can be presented as below [55]:

9 $$C_{s} { = }k_{f} {\text{C}}_{e}^{nf}.$$

The linearized form of the equation can be written as:

10 $$\ln C_{s} {\text{ = ln}}k_{f} { + }nf\ln {\text{C}}_{e}.$$

The coefficient n_f_ is a characteristic constant that describes sorption intensity, while the parameters C_s_, C_e_ and k_f_ represent the adsorbed amount (mmol g^−1^), concentration (residual) at equilibrium (mmol L^−1^) and the sorption capacity of sorbent (mmol g^−1^), respectively. All the k_f_ values decreased when temperature is increasing.

#### Adsorption thermodynamics

The study of thermodynamic parameters like equilibrium constants (K_c_), the standard enthalpy (∆H^°^), the standard Gibbs free energy (∆G^°^) and the standard entropy (∆S^°^) of the adsorption process of TMP onto SiNP-Cu were studied using the following equations [56]:

11 $$\Delta G^{^\circ } = - {\text{RTlnK}}_{c}.$$

12 $${\text{K}}_{c} = \frac{{C_{s} }}{{C_{e} }} + \frac{{\Delta S^{^\circ } }}{R}.$$

13 $${\text{lnK}}_{c} = - \frac{{\Delta H^{^\circ } }}{RT} + \frac{{\Delta S^{^\circ } }}{R}.$$

The parameter C_s_ is representing the concentration of TMP adsorbed (mol L^−1^); Ce is describing the equilibrium concentration of TMP in solution (mol L^−1^) at a given specific temperature; while T is the studied solution temperature (K); and R is the ideal gas constant (8.314 J K^−1^ mol^−1^). To find the standard enthalpies (∆H°) of the TMP-SiNP-Cu adsorption, the Van’t Hoff equation was used and the plots of ln K_c_ versus 1/T were obtained. The list of thermodynamic parameters are shown in Table 4. From the Table, the value for the enthalpy is positive (1.63 kJ mol^−1^). At the same time, the plot for Van’t Hoff which represents the interactions of the drug with the surface of the adsorbents usually require energy and as seen this interaction is endothermic in nature. The obtained low adsorption enthalpy values caused due to a number of physical interactions which can be described by the electrostatic attractions and nonpolar characteristics interactions, the hydrogen bonding and bridging.

**Table 4:** The standard thermodynamic parameters for TMP adsorption onto SiNP-Cu

Thermodynamic				
Temperature (K)	K_c_	∆G° (kJ mol^−1^)	∆S° (J mol^−1^)	∆H° (kJ mol^−1^)
308	0.318	2.53	3.73	1.63
315	0.321	2.62		
320	0.336	2.71		

Besides that, other parameters like ion-exchange reactions between TMP molecules and the adsorbent structure (SiNP-Cu) [55–57]. The main possibility factor for the low enthalpy is the H-bond and the formation of Water Bridge between the groups of N or O in the TMP organic structure (Fig. 1) and the group of –NH that is present during the synthesis of SiNP-Cu helped to lower the value. For the standard entropy (∆S°) which was determined from the intercept of the Van’t Hoof plot (∆S°/R) is positive (3.73 Jmol^−1^ K^−1^). This shows that the degrees of freedom of adsorbed species are increasing. At the same time, all the values for ∆G° at various temperatures are also positive. The values of the ∆G° of the process for SiNP-Cu decreased with increases the temperature, which lead to assume that the process may be spontaneous at high temperatures.

### Theoretical results

#### DFT study

**Fig. 10:**
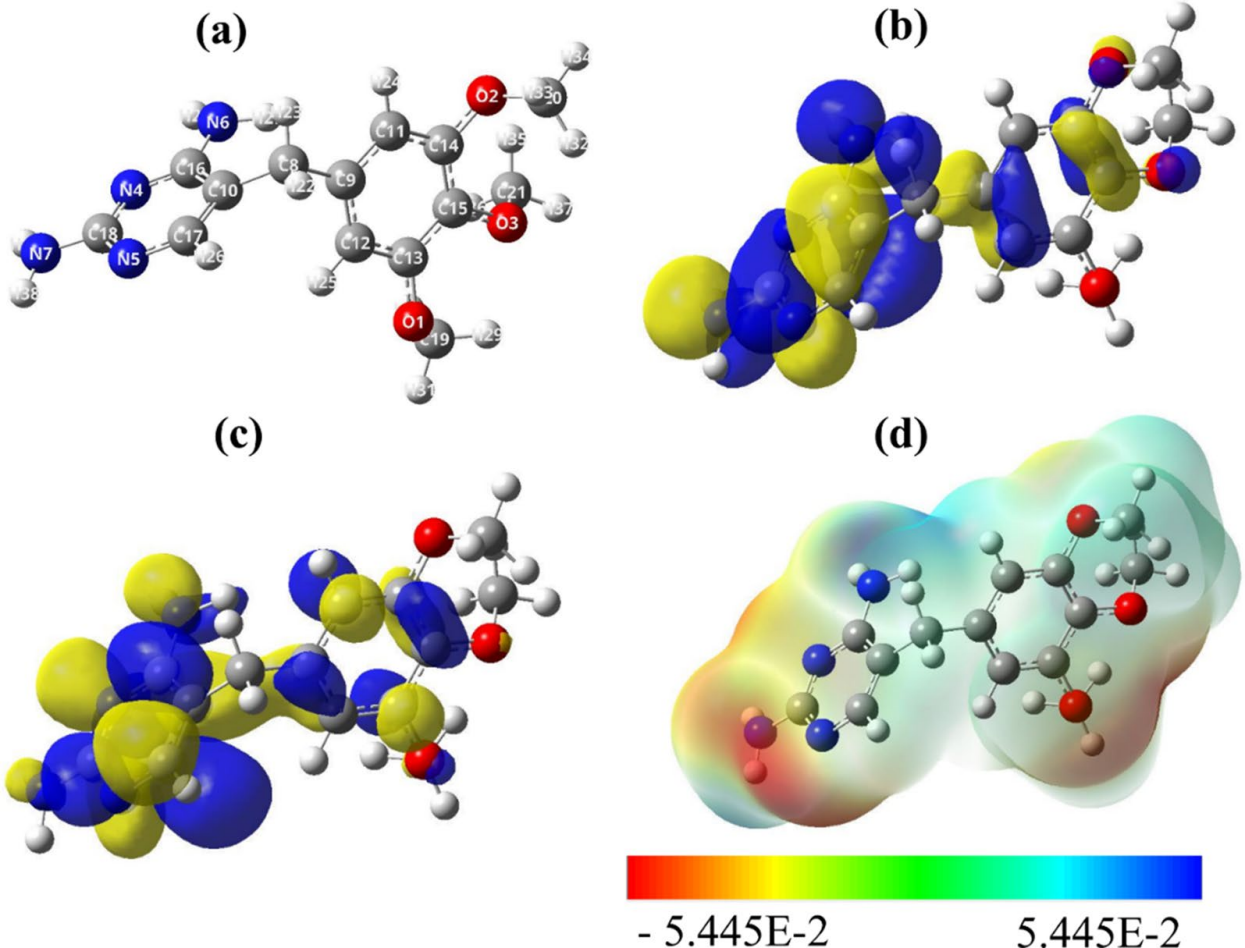
**a** optimized structure, **b** HOMO, **c** LUMO and **d** molecular electrostatic potential map (ESP) obtained using B3LYP/6–31 + G(d,p) level of theory in gas phase

As mentioned above, the main aim of this research study is the removal of the TMP from wastewater using SiNP (Fig. 10a). So that our discussion focuses on the results obtained in an aqueous solution. The optimized geometrical structures, the molecular electrostatic potential maps (ESP), and the distribution of HOMO orbital and LUMO orbital of the TMP compound are shown in Fig. 10b, c. We found that the distribution of the electron cloud in these two orbitals was mainly concentrated on most of the entire moiety of the molecule. In particular, we found that the HOMO orbital was more distributed on the N atom and the delocalized π-electrons of the pyrimidine and the aromatic benzene ring. This finding suggests that the N atoms and the delocalized π-electrons are responsible to donate the electrons to interact with the SiNP surface. On the other hand, we also found that the LUMO was found more distributed on the C atoms, suggesting that the C atoms are the centers responsible to accept an electron from the SiNP. These findings were also confirmed by monitoring the ESP map (see Fig. 10d). The small value of the energy gap indicates the high reactivity and the ease of the adsorption process of the TMP on the SiNP surface (Table 5). As in known, in the process of adsorption of TMP by SiNP, the electron charge is transferred from the TMP toward the SiNP surface, and this result is in agreement with the positive values charge transfer maximum parameter (∆N) [58]. The positive value of ∆N proves that the TMP has a donor electron effect and is a donor electron (Table 5). Furthermore, the chemical electronic potential of the TMP is negative and it means that the TMP is stable and it is not decomposing spontaneously into the elements and compounds are made up of them. The energy gap of TMP is quite high. The dipole moment of TMP (4.97 Debye) is higher than that of water (1.82 Debye) and it means that TMP is able to expel water from the SiNP surface (Table 5).

**Table 5:** Quantum global reactivity descriptors of the TMP molecule

	Gas phase	Aqueous solution
E_total_ (hartree)	− 989.054	− 989.071
Volume (bohr^3^/mol)	2174.001	2561.223
Dipole moment μ^*^ (Debye)	4.05	4.97
*E*_*HUMO*_ (eV)	− 5.883	− 6.154
*E*_*LUMO*_ (eV)	− 0.637	− 0.897
*E*_*HOMO− 1*_ (eV)	− 6.346	− 6.522
*E*_*HOMO− 2*_ (eV)	− 6.590	− 6.803
*Energy gap ΔE (eV)*	*5.245*	*5.258*
*Hardness (η) (eV)*	*2.623*	*2.629*
*Hyper-hardness (γ)*	*4.782*	*4.890*
*Softness S (eV*^*−1*^*)*	*0.381*	*0.380*
*Chemical potential (*$\pi$*) (eV)*	*− 3.260*	*− 3.525*
*Electrophilicity (ω) (eV)*	*2.026*	*2.364*
*Maximum charge transfer (ΔN)*	*1.243*	*1.341*

**Fig. 11:**
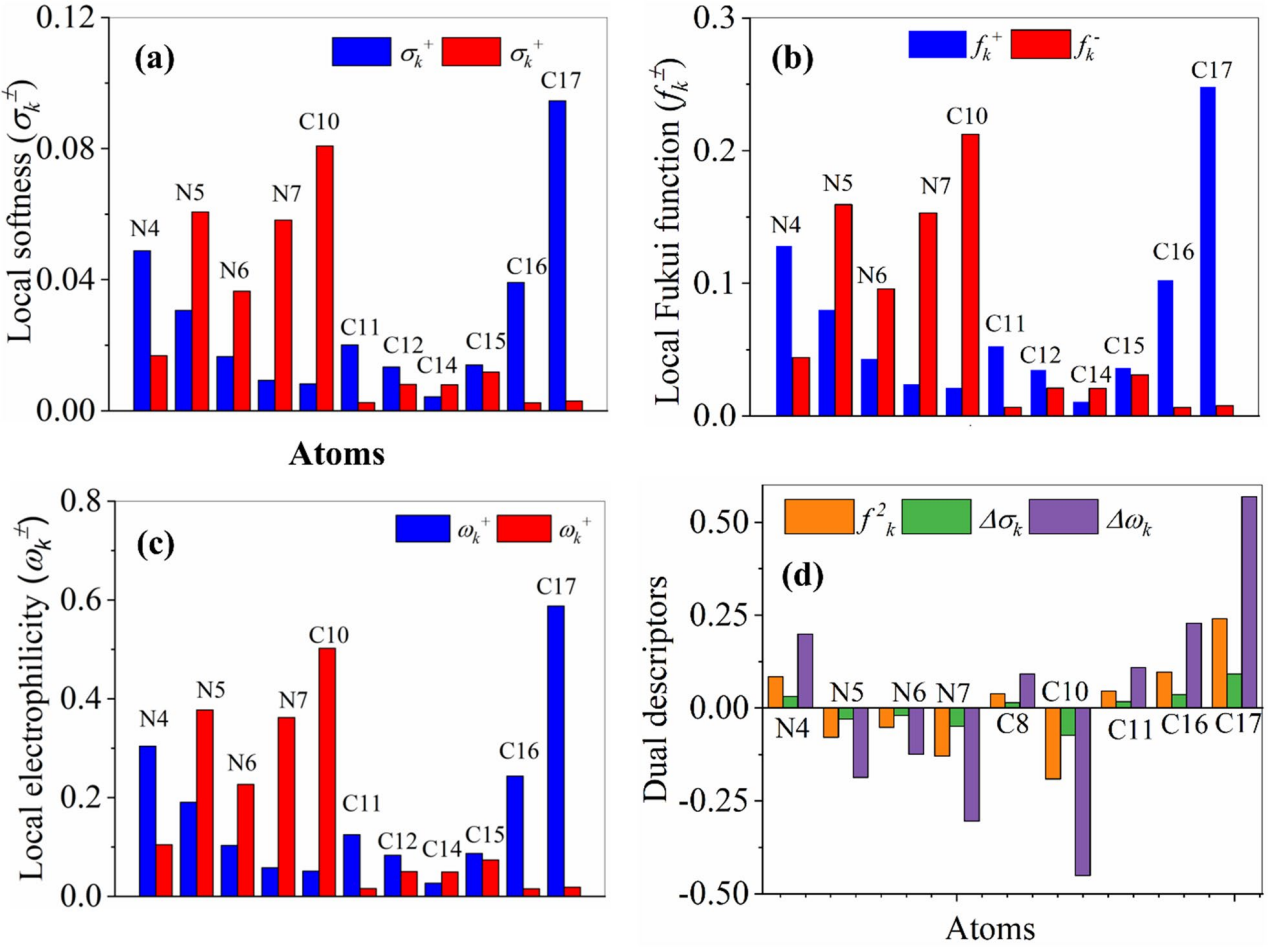
Graphical representation of the LRDs, **a** ${f}_{k}^{\pm }, \mathbf{b} {\sigma }_{k}^{\pm }$, **c **${\omega \sigma }_{k}^{\pm }$, and **d** the local dual descriptors, (${f}_{k}^{2}$, *Δσ*_*k*_ and *Δω*_*k*_) based on Fukui Functions of the TMP compound obtained using B3LYP/6–31 + G(d,p) level of theory in aqueous solution (the atom-numbering is in correspond with Fig. 1a)

The full set of the NBO charges, LRDs and DDs obtained at B3LYP/6–31 + G (d,p) level of theory in aqueous solution are listed in Table 5 of the electronic supplementary information (ESI). Figure 11a–c shows the graphical representation of the LRDs (${f}_{k}^{\pm }, {\sigma }_{k}^{\pm }$ and ${\omega \sigma }_{k}^{\pm }, \mathrm{repectively}$) of the TMP compound**.** Our computed results suggest that the highest nucleophilic attack $\left({f}_{k}^{+}\right)$ are found on C17, N4 and C16, whereas, the highest electrophilic attack $\left({f}_{k}^{-}\right)$ are found on C10, N5, N7 and N6, see (Fig. 11a). As known, the most nucleophilic sites in investigated compounds have the highest value of ${\sigma }_{k}^{-} \mathrm{and} {\omega }_{k}^{-}$, while the highest value of ${\sigma }_{k}^{+} \mathrm{and} {\omega }_{k}^{+}$ reveals the most electrophilic site in TMP compound [59], see panels b and c of Fig. 11. For numerical results, see Table 5 of the ESI. Consequently, similar conclusions can also be marked by following the results of the local softness and the local electrophilicity.

Figure 11d shows the graphical representations of the dual reactivity descriptors of TMP compound obtained using B3LYP/6–31 + G(d,p) level of theory in aqueous solution. Full set of numerical results of the DDs can be followed in Table 5 of ESI. A close inspection of the figure and Table 5 reveals that the most active sites, with ${f}_{k}^{2}, \Delta {\sigma }_{k} \mathrm{and} \Delta {\omega }_{k}<0$, that are responsible to donate electron to SiNP surface are C10, N7, N5 and N6, whereas the most active sites, with ${f}_{k}^{2}, \Delta {\sigma }_{k} and \Delta {\omega }_{k}>0$, that are responsible to accept an electron from SiNP surface are C17, C16 and N4. These results agree with the results obtained by HOMO, LUMO and ESP maps

#### Monte Carlo (MC) and Molecular Dynamic (MD) simulation

**Fig. 12:**
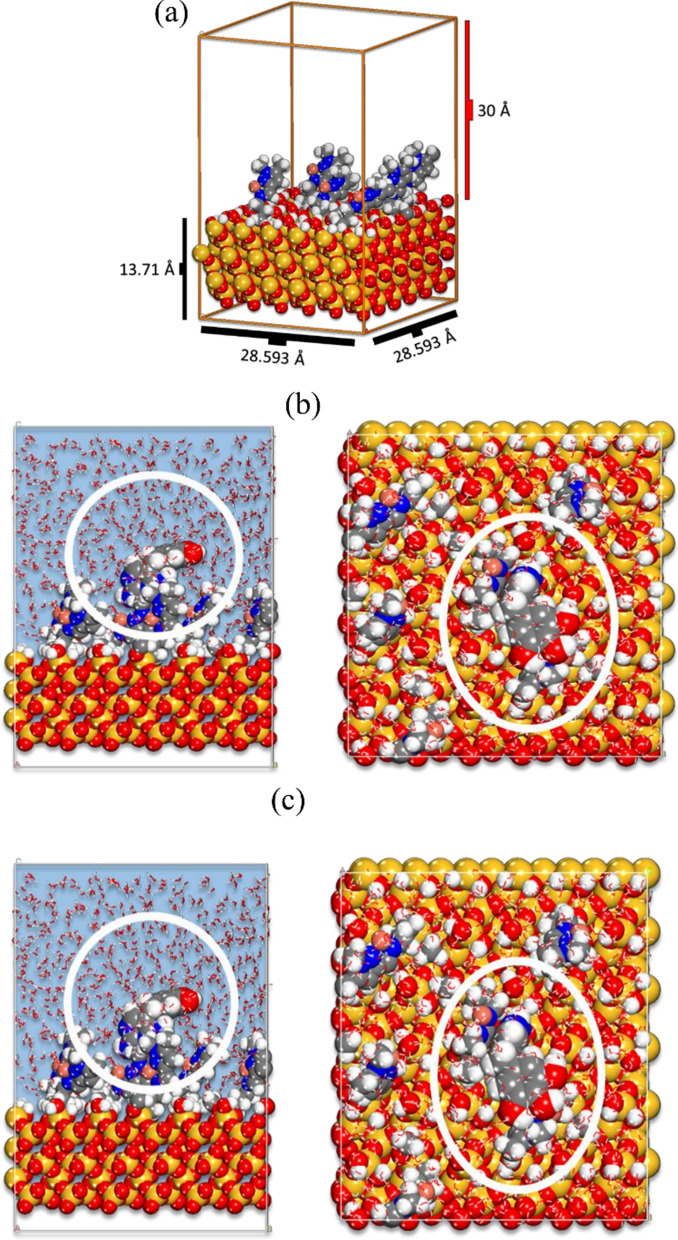
**a** The size of the simulation box containing the modified silica surface and the vacuum layer, **b** MC poses of the lowest adsorption configurations for onto Modified silica surface and **c** MD lowest energy configurations of TMP molecule interaction onto Modified silica surface

The interaction between the modified silica surface and the TMP molecule was investigated using a large number of randomized Monte Carlo steps (configurations). The MD computations continue to employ the lowest energy geometry as provided by the MC. The simulation is performed under Periodic Boundary Conditions using the cell with dimensions presented in Fig. 12a. In the simulations the modified silica surface box is filled with 1 TMP and 650 water molecules. Prior to the MD stage, the geometry was optimized using the Force module built into the Biovia software (tolerance for energy convergence of 1 × 10–5 kcal/mol; atom-based summation method for both electrostatic and van der Waals interactions with a cutoff distance of 15.5, a spline width of 1, and a 0.5 buffer; atom-based summation method for both electrostatic and van der Waals interactions with a cutoff distance of 15.5, a s MD was done at 25 °C with a 1 ns simulation duration using the Constant volume/constant temperature (NVT) canonical ensemble (using a 1 fs time step) [59]. The Berendsen thermostat maintains the T control. Calculations for MC and MD are performed using the Universal force field [60].

The lowest energy configurations for the silica surface and the TMP molecule are shown in Fig. 12b. The measurable verdict of the interaction between TMP molecule and the modified silica surface is calculated using the following equation:

$${E}_{ads}={E}_{total}-\left[{E}_{\mathrm{TMP}}+{E}_{\mathrm{Modified silica}}\right]$$

where: E_total_ is the total energy of the system as a result of Modified Silica surface and the TMP molecule interaction; ${E}_{\mathrm{Cu}\left(\mathrm{II}\right)or Pb\left(II\right)ions}$ and ${E}_{\mathrm{Modified silica}} is$ system energy in the absence and presence of TMP molecule. MC calculations yield the lowest energy pose after a considerable number of randomized configurations. The Monte Carlo simulations (Fig. 12b) show that the TMP molecule adsorbs extensively on the modified silica surface, which is consistent with the experimental findings. The adsorption’s negative value indicates the adsorption process' spontaneity on this adsorbent [61]. Figure 11a–d in the ESI depicts the energies during the attendance of the lowest energy position, The graph shows temperature control from MD during the interaction of the TMP molecule onto modified silica surface, and the interaction energy of the TMP molecule onto modified silica surface during MD, respectively.

The distribution of the adsorption energies as shown in Fig. 12b are in range − 5 to − 95 kcal/mol depending on the contact configurations among TMP and modified silica surface. MD is primarily concerned with monitoring the overall dynamics of the process. The small temperature drift on the graph in Fig. 12c shows that the equilibrium configuration has been reached. Figure 12c shows the lowest equilibrium energy structure for the TMP molecule interaction with modified silica surface obtained from MD. The interaction (adsorption) energy during the MD is assessed at each time elapse and is presented in Fig. 12c and descriptive statistics in Table 6. Relative high adsorption energies are consistent with experimental findings. The negative values of the adsorption energies indicate the spontaneity of the adsorption process.

**Table 6:** Statistics of the interaction energy of the TMP molecule onto modified silica surface during MD (energy values are in kcal/mol)

Molecule	Mean	Minimum	Median	Maximum
TMP	− 15.48	− 3.78	− 16.51	− 29.58

## Regeneration of adsorbent

**Fig. 13:**
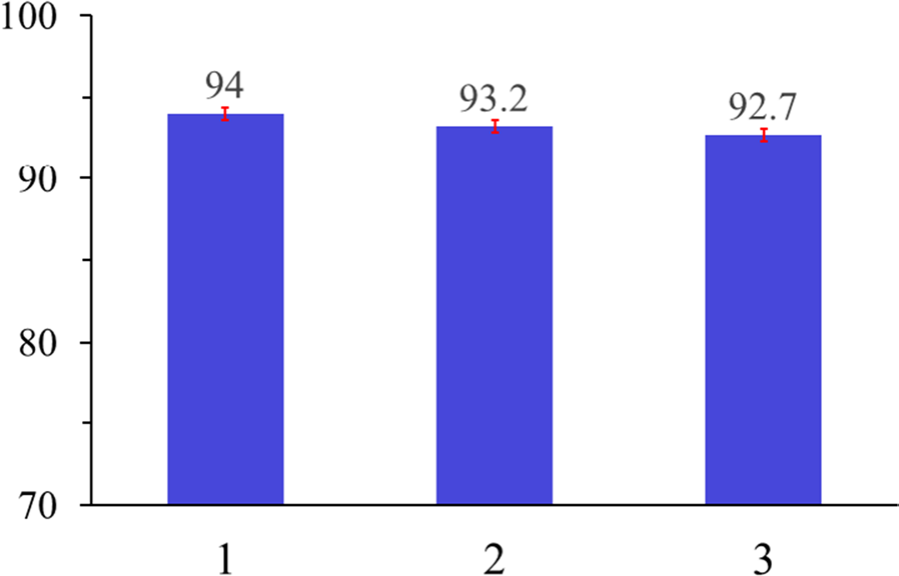
SiNP-Cu regeneration using 1 N HCl

As shown in Fig. 13, the effect of adsorbent recovery on adsorption of TMP on SiNP-Cu The difference in %removal between the first and second uses of SiNP-Cu adsorbent for removal of TMP was very small, 0.85% loss of efficiency, and it was also lower by 1.38% for the third use.

## Conclusion

Recently, several research studies for pharmaceutical and personal care products (PPCPs) have been occurring around the world because of their severe distribution, continuous release, and huge effects on wildlife in the environment have been studied for their removal from wastewater. This research used 1,5-Dimethyl-1H-pyrazole-3-carbaldehyde, which was fixed on the silica surface after many treatments and modifications, including 3-aminopyltrimethoxysilane, and then refluxed with copper nitrate to produce SiNP-Cu which was used as adsorbent for the removal of TMP. The adsorbent demonstrated a high percentage removal of TMP, reaching more than 94 percent. FT-IR spectra, nitrogen adsorption–desorption isotherm, BET surface area, B.J.H. pore diameters, thermogravimetric analysis (TGA), and scanning electron microscopy (SEM) were used to characterize the newly synthesized material. The novel chelating surface is chemically and thermally stable. By immersing the sample in 1 N HCl for a few minutes. The sorbent was regenerated three times and the extraction % did not change significantly. The absorption mechanism was highly pH dependent and followed Freundlich and pseudo second order models. The adsorption process was not spontaneous.

The global reactivity indics prove that TMP is stable and it can be removed from wastewater using SiNP surface. The results of the local reactivity indices concluded that the active centers for the adsorption process are the nitrogen atoms and the π-electrons of the pyrimidine and benzene rings. Furthermore, the positive value of the maximum charge transfer number (*ΔN*) proves that TMP has a great tendency to donate electrons to SiNP surface during the process of adsorption.

The relative high adsorption energies obtained by MD simulation study are consistent with experimental findings. The negative values of the adsorption energies indicate the spontaneity of the adsorption process.

In general, the modified SiNP-Cu showed excellent TMP removal with more than 94% in basic medium.

## Data Availability

The majority of the data used to support the findings of this study are included within the article. Other data are available from the corresponding author upon request.
